# Supplementary material for: Identification of Antimicrobial Compounds from Sandwithia guyanensis-Associated Endophyte Using Molecular Network Approach
Source: Plants (Basel). 2019 Dec 29;9(1):47. doi: 10.3390/plants9010047 (PMC7020175; doi:10.3390/plants9010047)

Identification of Antimicrobial Compounds from *Sandwithia guyanensis*-Associated Endophyte Using Molecular Network Approach

Phuong-Y Mai [1], Marceau Levasseur [1], Didier Buisson [2] , David Touboul [1] and Véronique Eparvier [1,*]

[1] Paris-Saclay CNRS ICSN, Institut de Chimie des Substances Naturelles UPR 2301, Université 91198 Gif-sur-Yvette; maiphuongy@cnrs.fr ; marceau.levasseur@cnrs.fr ; david.touboul@cnrs.fr ; veronique.eparvier@cnrs.fr

[2] Museum National d'Histoire Naturelle, Molécules de Communication et Adaptation des Micro-organismes, UMR 7245 CNRS/MNHN, Sorbonne Université, Paris Cedex 05 ; didier.buisson@mnhn.fr

* Correspondence: veronique.eparvier@cnrs.fr; Tel.: +33-169-823-679 (V.E.)

SUPPLEMENTARY DATA

Table S1: Antimicrobial and cytotoxic activities of endophyte extracts (endophytes isolated from latex are notified in bold from bark in italic the overall are isolated from leaves)

	Endophytic extracts	MIC* on MRSA**	MIC on *C. albicans*	MIC on *T. rubrum*	MRC5 (% of cell viability)	
		µg/mL	µg/mL	µg/mL	10 µg/mL	1 µg/mL
Individual 1	BSNB-SG1.1	128	> 256	> 256	-	-
	BSNB-SG1.2	256	256	**8**	100.5 ± 2.4	100.3 ± 2.4
	BSNB-SG1.3	256	> 256	> 256	-	-
	BSNB-SG1.4	128	256	> 256	-	-
	BSNB-SG1.5	256	128	256	-	-
	BSNB-SG1.6	**64**	128	256	-	-
	BSNB-SG1.7	256	256	> 256	-	-
	BSNB-SG1.8	**64**	> 256	> 256	-	-
	BSNB-SG1.9	256	256	256	-	-
	BSNB-SG1.10	256	128	256	-	-
Individual 2	BSNB-SG2.1	**64**	**64**	256	25 ± 1	55 ± 2
	BSNB-SG2.2	256	> 256	**8**	97.5 ± 1.5	102.3 ± 1.5
	BSNB-SG2.3	128	256	> 256	-	-
	BSNB-SG2.4	128	> 256	> 256	-	-
	BSNB-SG2.5	128	> 256	**64**	99.4 ± 2.6	104.1 ± 2.6
	BSNB-SG2.6	128	256	**8**	52.3 ± 4.5	95.6 ± 4.5
	BSNB-SG2.7	256	32	256	103.3 ± 3.7	101.9 ± 3.7
	BSNB-SG2.8	256	256	**8**	88.3 ± 2.3	100.6 ± 2.3
	BSNB-SG2.9	**64**	256	**8**	48 ± 2	82 ± 3
	BSNB-SG2.10	128	> 256	> 256	-	-
	BSNB-SG2.11	128	> 256	> 256	-	-
	BSNB-SG2.12	128	256	> 256	-	-
	BSNB-SG2.13	256	> 256	128	-	-
	BSNB-SG2.14	256	> 256	> 256	-	-
	**BSNB-SG2.15**	256	> 256	> 256	-	-
	**BSNB-SG2.16**	**64**	**64**	128	53 ± 1	63 ± 2
	**BSNB-SG2.17**	256	> 256	> 256	-	-
	**BSNB-SG2.18**	128	> 256	> 256	-	-
	**BSNB-SG2.19**	**64**	256	> 256	-	-
	**BSNB-SG2.20**	256	> 256	> 256	-	-
Individual 3	*BSNB-SG3.1*	256	> 256	> 256	-	-
	BSNB-SG3.2	128	256	> 256	-	-
	BSNB-SG3.3	256	256	256	-	-
	BSNB-SG3.4	256	> 256	> 256	-	-
	**BSNB-SG3.5**	256	256	> 256	-	-
	**BSNB-SG3.6**	128	> 256	256	-	-
	**BSNB-SG3.7**	**16**	**128**	> 256	65 ± 2	92 ± 3
	**BSNB-SG3.8**	**32**	> 256	> 256	55 ± 3	86 ± 4
	**BSNB-SG3.9**	> 256	> 256	> 256	-	-
	**BSNB-SG3.10**	128	**8**	256	9.7 ± 2.8	7.3 ± 2.8
	**BSNB-SG3.11**	**32**	**8**	**8**	29 ± 2	82 ± 2
	**BSNB-SG3.12**	256	> 256	> 256	-	-

- : not determined,
* : methicillin resistant *Staphylococcus aureus*
** : Minimum inhibitory concentration

Table S2: ^{1}H NMR data of compounds **1**, **2** and **3** in CD_3CN

Pos.		**Compound 1** δ_H, m (*J* in Hz)	**Compound 2** δ_H, m (*J* in Hz)	**Compound 5** δ_H, m (*J* in Hz)	Pos.		**Compound 3** δ_H, m (*J* in Hz)	**Compound 4** δ_H, m (*J* in Hz)
**Thr**	1	4.55, d (8.8)	4.56, d (8.2)	4.56, dd (9.1, 1.1)	**Thr**	1	4.55, d (9.0)	4.54, dd (8.3, 1.1)
	2	5.41, q (6.7)	5.43, q (6.8)	5.42, m		2	5.42, q (6.4)	5.41, qd (6.1, 1.1)
	3	1.28, m	1.28, m	1.29, m		3	1.29, m	1.29, m
	NH	7.10, d (9.6)	7.05, d (8.9)	7.07, d (9.2)		NH	7.03, d (8.9)	7.02, d (8.9)
**Ser**	1	4.32, m	4.34, m	4.36, m	**Ser**	1	4.34, m	4.34, m
	2/2’	3.75, dd (*J* = 15.8, 5.5)	3.78, m	3.78, m		2/2’	3.78, m	3.77, m
	NH	7.35, d (7.8)	7.26, d (7.5)	7.28, d (7.7)		NH	7.30, d (6.9)	7.27, d (6.7)
**Ser**	1	4.36, m	4.37, m	4.36, m	**Ser**	1	4.34, m	4.34, m
	2/2’	3.69, d (5.8)	3.70, d (5.3)	3.71, d (5.5)		2/2’	3.71, d (5.5)	3.71, t (5.4)
	NH	6.98, d (7.8)	6.93, d (7.8)	6.96, d (7.2)		NH	6.95, m	6.96, d (7.4)
**Val**	1	4.14, t (7.7)	4.16, t (8.8)	4.16, t (8.9)	**Val**	1	4.19, m	4.18, m
	2	2.08, m	2.13, m			2	2.13, m	2.35, m
	3/3’	0.94, m	0.96, m			3/3’	0.96, m	0.95, m
	NH	7.15, d (8.9)	7.11, d (9.0)	7.11, d (9.0)		NH	7.12, d (9.2)	7.10, d (8.7)
**Ile**	1	4.23, t (6.8)	4.23, t (7.3)	4.24, t (6.8)	**Val**	1	4.19, m	4.18, m
	2	1.81, m	1.82, m	1.82, m		2	2.06, m	2.31, m
	3	0.91, m	0.91, m	0.91, m		3	0.96, m	0.95, m
	4	1.20, m	1.77, m	1.21, m		3’	0.96, m	0.95, m
	4’	1.45, m	1.80, m	1.51, m		NH	6.95, m	6.96, d (7.4)
	5	0.93, m	0.92, m	0.96, t (5.63)				
	NH	6.96, d (7.8)	6.96, d (7.5)	6.96, d (7.2)				
**Acyl**	2	2.34, m	2.34, m	2.36, m	**Acyl**	2	2.35, m	2.34, m
	3	1.64, m	1.66, m	1.71, q (7.45)		3	1.67, m	1.66, m
	4	1.26, m	1.32, m	1.29-1.38, m		4	1.31, m	1.31, m
	5	1.23-1.34, m	1.29-1.37, m	5.42, m		5	1.30-1.37, m	1.28-1.34, m
	6	1.23-1.34, m	1.29-1.37, m	5.42, m		6	1.30-1.37, m	1.28-1.34, m
	7	1.23-1.34, m	1.29-1.37, m	1.29-1.38, m		7	1.30-1.37, m	1.28-1.34, m
	8	1.23-1.34, m	1.29-1.37, m	1.29-1.38, m		8	1.30-1.37, m	1.28-1.34, m
	9	1.23-1.34, m	1.21, m	1.29-1.38, m		9	1.30-1.37, m	1.28, m
	10	1.23-1.34, m	0.93, m	1.29-1.38, m		10	1.30-1.37, m	0.95, m
	11	1.29, m	-	1.29, m		11	1.34, m	-
	12	0.92, m	-	0.96, m		12	0.93, m	-

Table S3: ^{13}C NMR data of compounds **1**, **2** and **3** in CD_3CN

Pos.		Compound 1 δ_C	Compound 2 δ_C	Compound 5 δ_C	Pos.		Compound 3 δ_C
**Thr**	C=O	171.6	170.9	170.0	**Thr**	C=O	
	1	57.4	56.8	55.6		1	57.1
	2	72.2	71.2	71.2		2	71.9
	3	19.7	19.6	18.0		3	
**Ser**	C=O	173.0	172.2	171.4	**Ser**	C=O	
	1	56.1	55.2	54.9		1	55.6
	2/2’	62.3	61.6	62.1		2/2’	61.8
	NH					NH	
**Ser**	C=O	173.1	172.6	171.4	**Ser**	C=O	
	1	55.8	55.3	54.9		1	55.6
	2/2’	56.1	62.5	61.1	2/2’		62.6
**Val**	C=O	172.9	172.3	171.5	**Val**	C=O	
	1	58.9	59.3	58.5		1	59.6
	2	30.6	30.1	29.4		2	30.2
	3/3’	19.7	19.1	18.3		3 and 3’	19.3
**Ile**	C=O	172.6	172.0	171.0	**Val**	C=O	
	1	60.1	58.1	57.8		1	59.6
	2	37.9	37.2	36.6		2	31.4
	3	12.2	11.5	14.7		3/3’	19.3
	4/4’	26.8	26.8	25.8			
	5	20.2	15.4	10.5			
**Acyl**	1	175.6	174.9	171.3	**Acyl**	1	174.0
	2	37.5	36.8	35.6		2	37.0
	3	27.3	26.5	25.8		3	27.1
	4	33.4	30.8	26.8		4	33.3
	5	30.6-31.1	28.8-31.2	129.4		5	29.0-32.1
	6	30.6-31.1	28.8-31.2	130.6		6	29.0-32.1
	7	30.6-31.1	28.8-31.2	25.8-36.9		7	29.0-32.1
	8	30.6-31.1	28.8-31.2	25.8-36.9		8	29.0-32.1
	9	30.6-31.1	26.5	25.8-36.9		9	29.0-32.1
	10	30.6-31.1	11.8	25.8-36.9		10	29.0-32.1
	11	24.3	-	22.6		11	24.0
	12	15.0	-	14.4		12	15.6

Figure S1: Global Molecular Networks of 42 extracts strain from *S. guyanenis*. Each endophyte extract is depicted by one color.

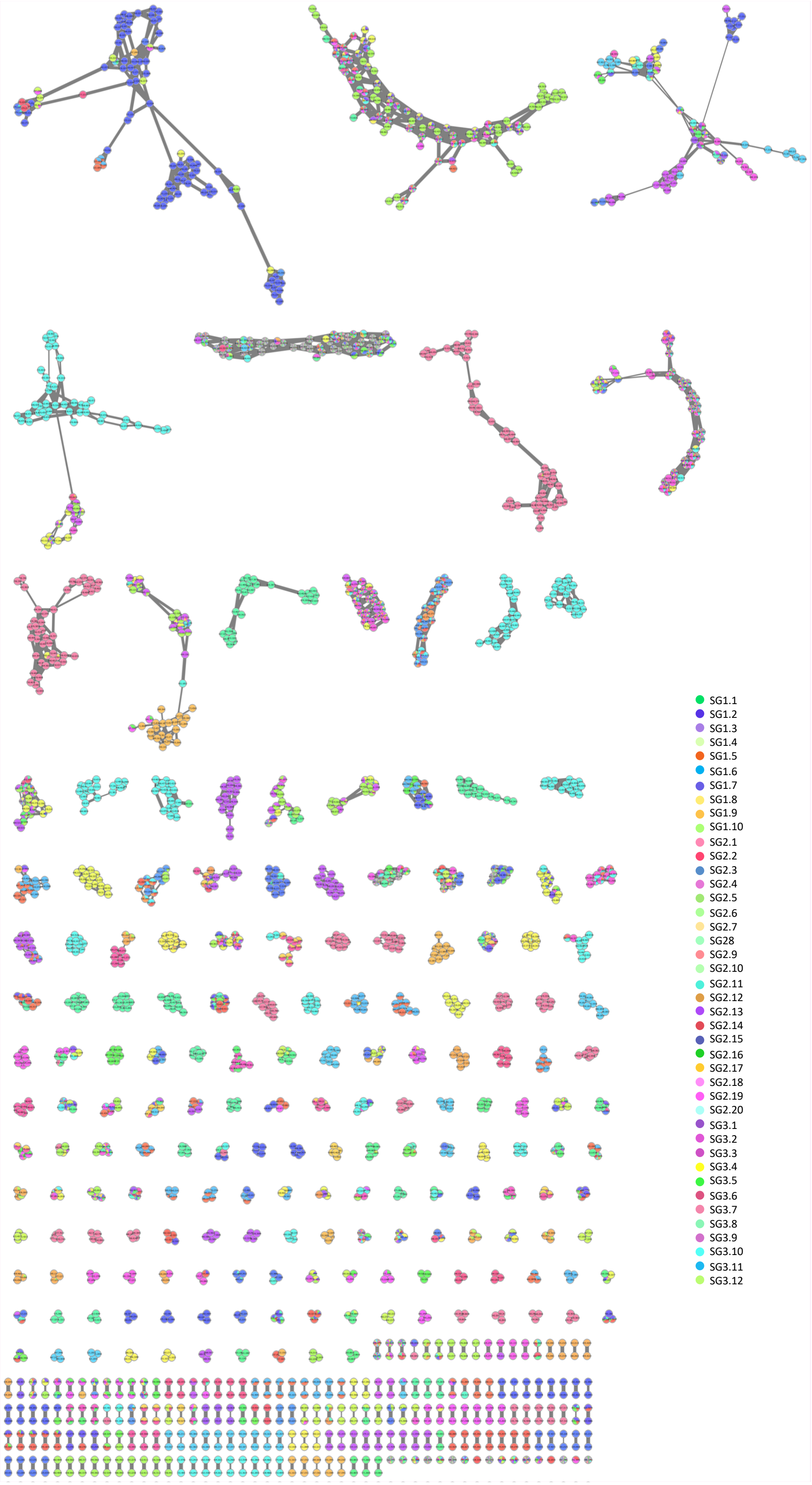

Supplementary Figure S2: Global Molecular Networks of 42 extracts strain from *S. guyanenis*. The clusters from active extracts on *Candida albicans* are shown in green.

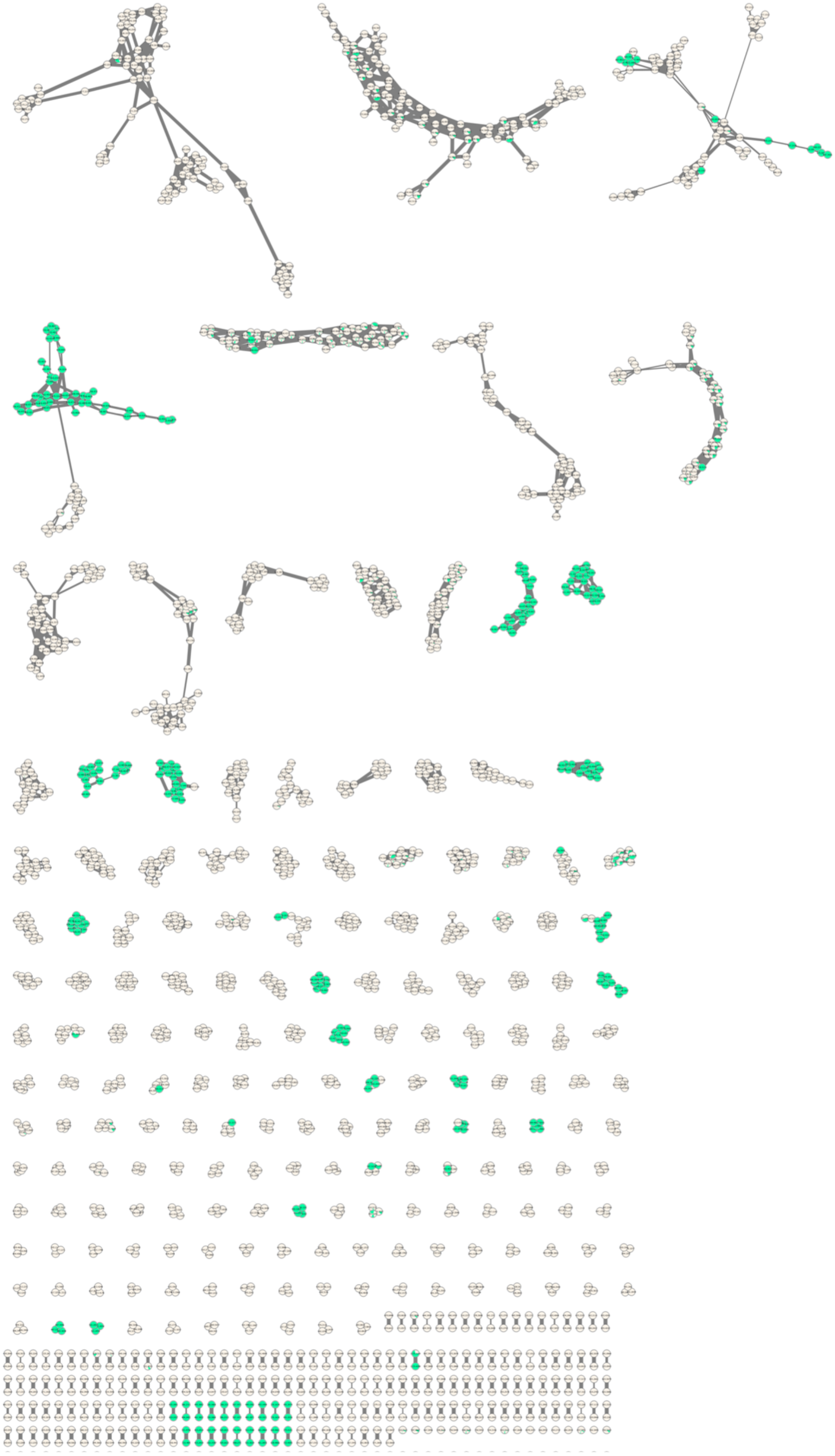

Supplementary Figure S3: Global Molecular Networks of 42 extracts strain from *S. guyanenis*. The clusters from active extracts on *Trichophyton rubrum* are shown in blue.

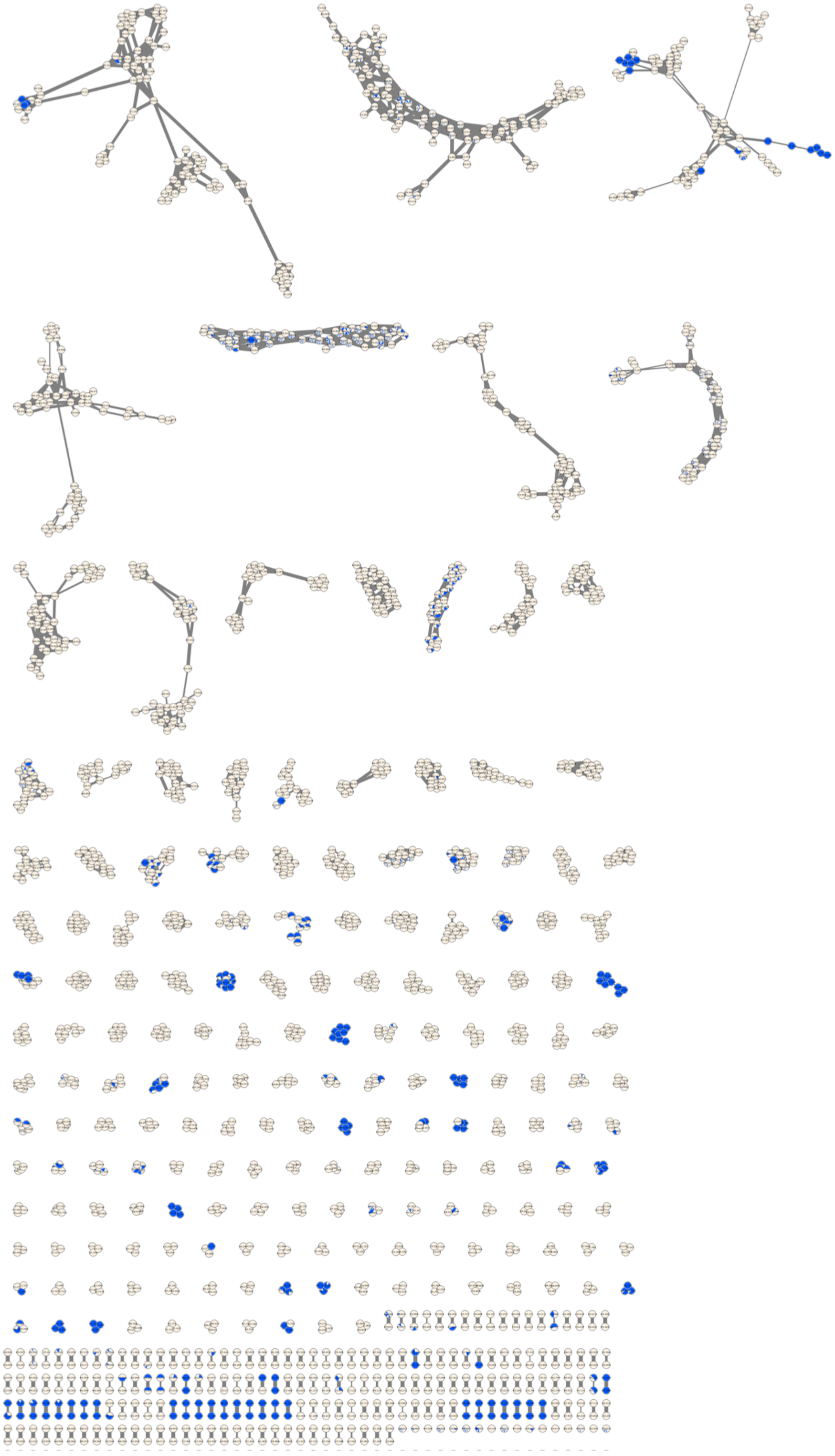

Supplementary Figure S4: Global Molecular Networks of 42 extracts strain from *S. guyanenis*. The clusters of endophytes extracts isolated from plant latex are shown in yellow.

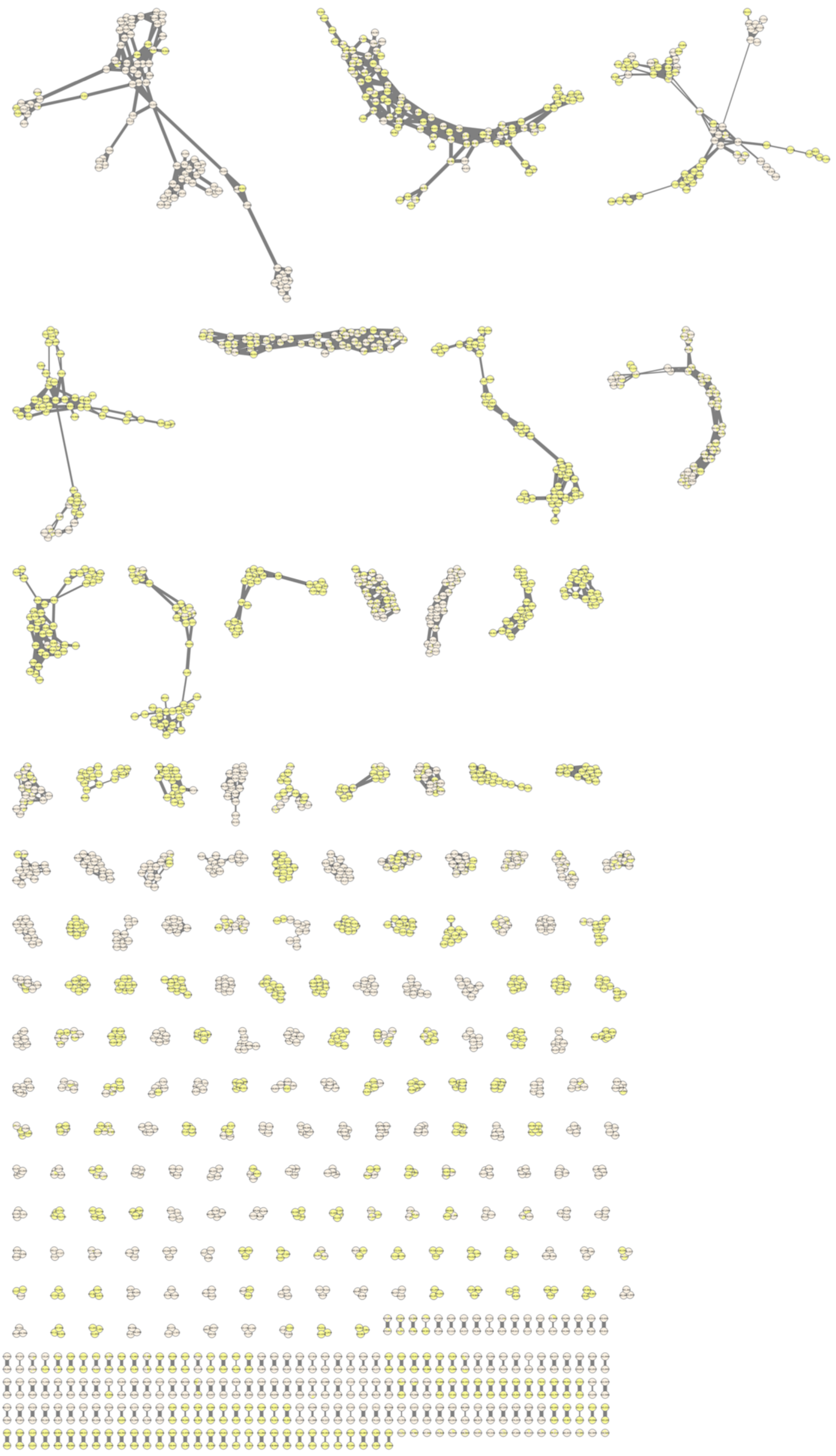

Supplementary Figure S5: Mass spectrum of compound **1**

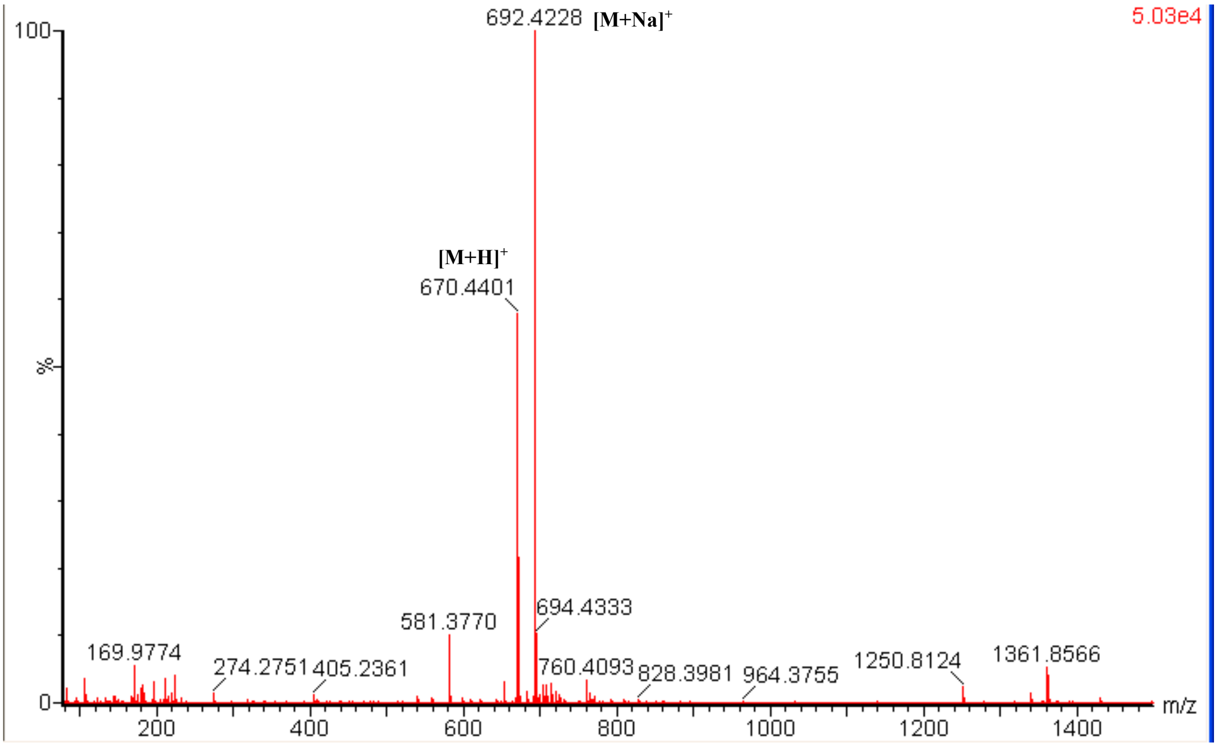

Supplementary Figure S6: Mass spectrum MS/MS of compound **1** – A: The enlargement of ion peak at *m/z* 183.1706 relevant to the acyl chain

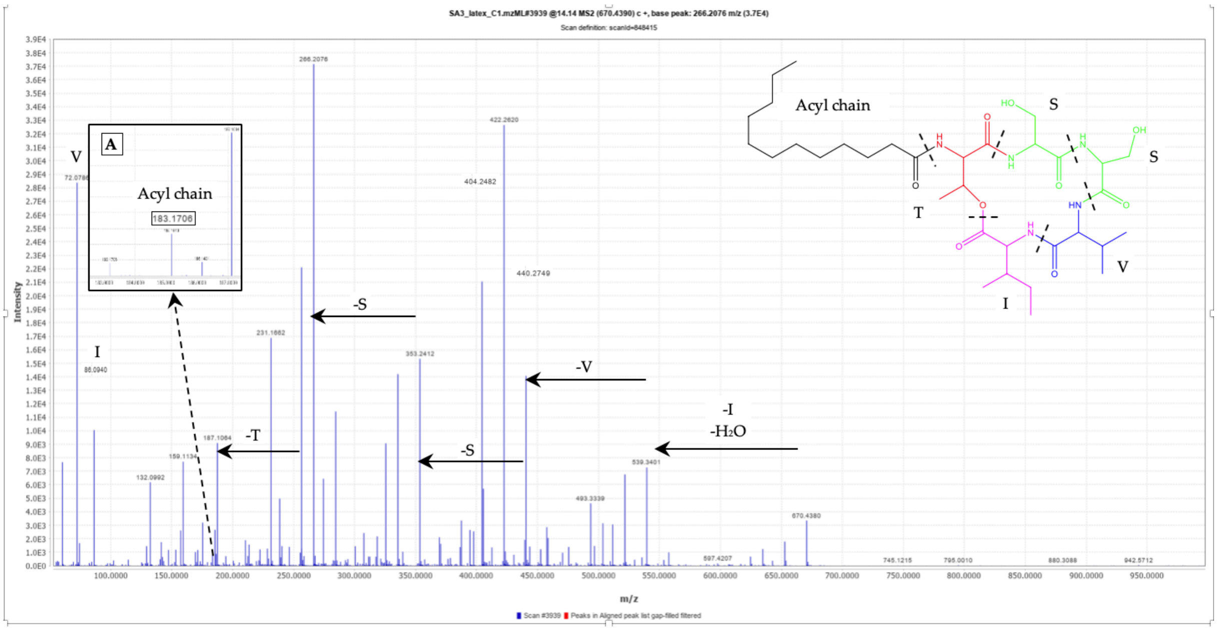

Supplementary Figure S7: ^{1}H NMR spectrum of compound **1** recorded at 500 MHz in CD_3CN

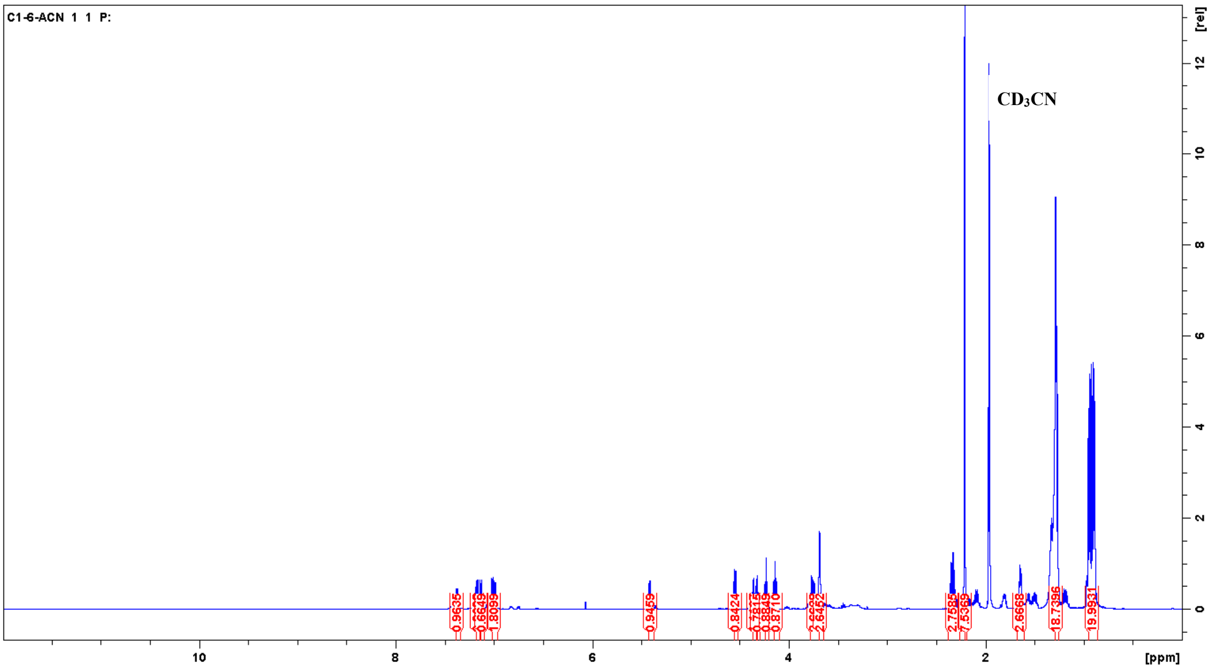

Supplementary Figure S8: ^{1}H-^{1}H COSY spectrum of compound **1** recorded at 500 MHz in CD_3CN

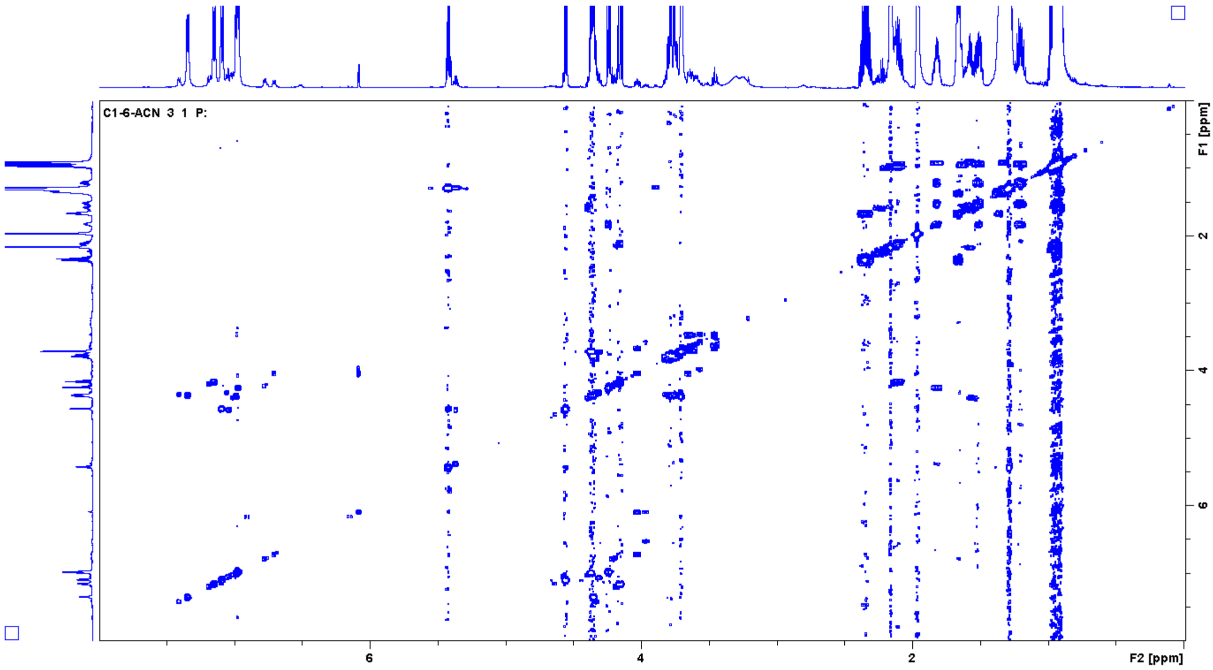

Supplementary Figure S9: ^{13}C NMR spectrum of compound **1** recorded at 125 MHz in CD_3CN

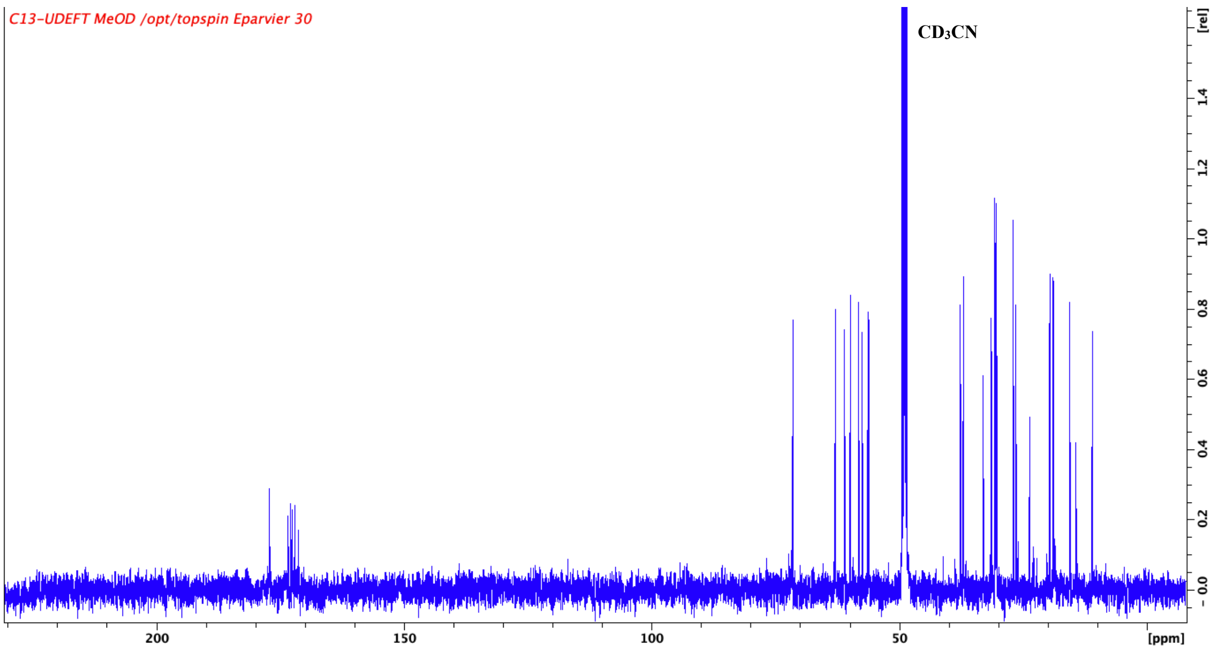

Supplementary Figure S10: ^1H-^{13}C HSQC spectrum of compound **1** recorded at 500 MHz in CD_3CN

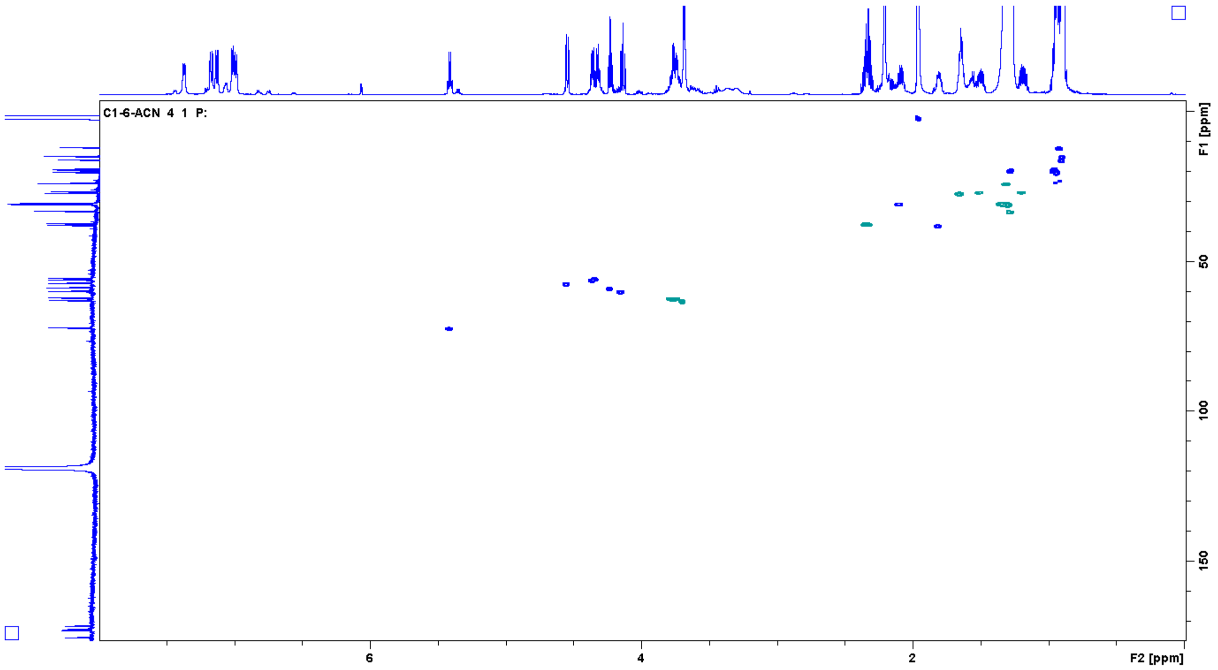

Supplementary Figure S11: ^{1}H-^{13}C HMBC spectrum of compound **1** recorded at 500 MHz in CD_3CN

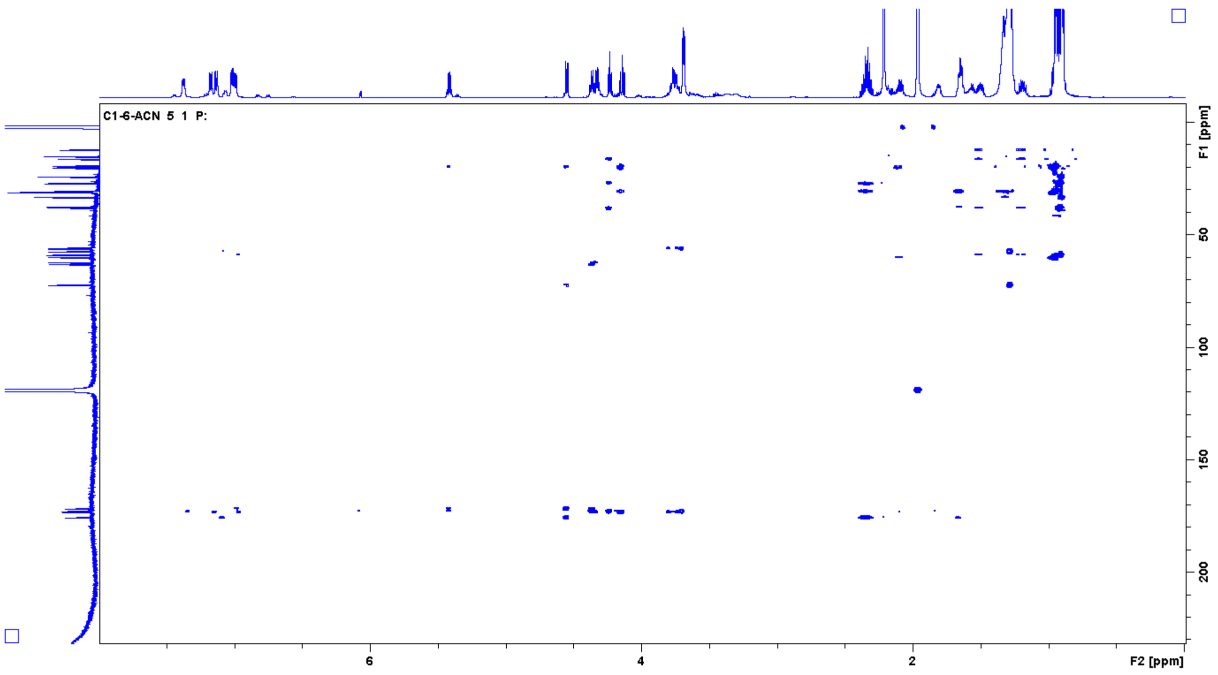

Supplementary Figure S12: DEPT spectrum of compound **1** recorded at 125 MHz in CD_3CN, which is shows CH/CH_3 with a negative phase and CH_2 with a positive phase.

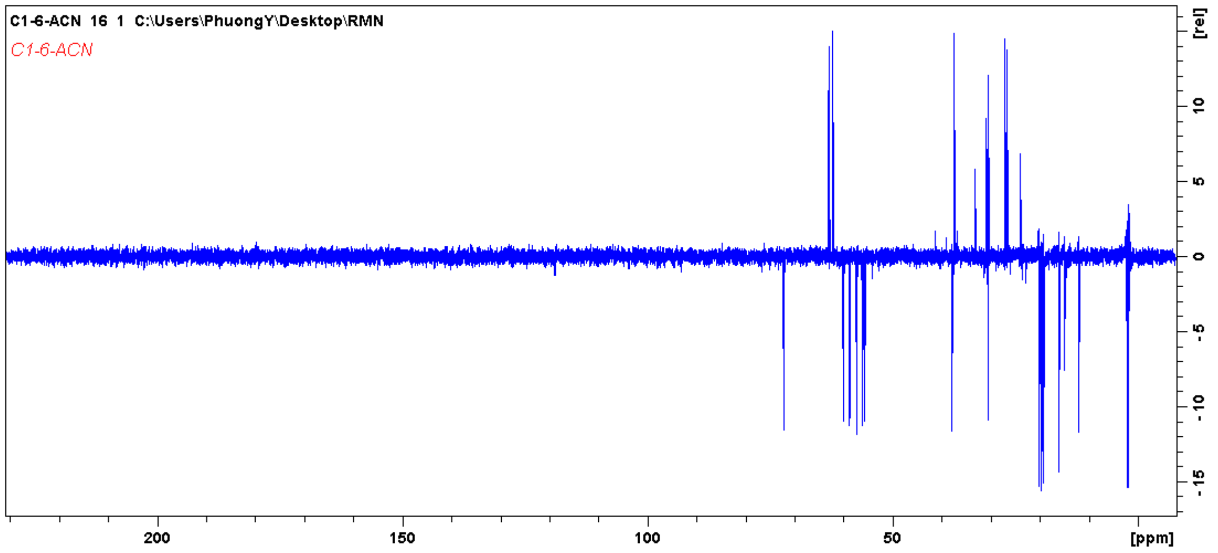

Supplementary Figure S13: Mass spectrum of compound **2**

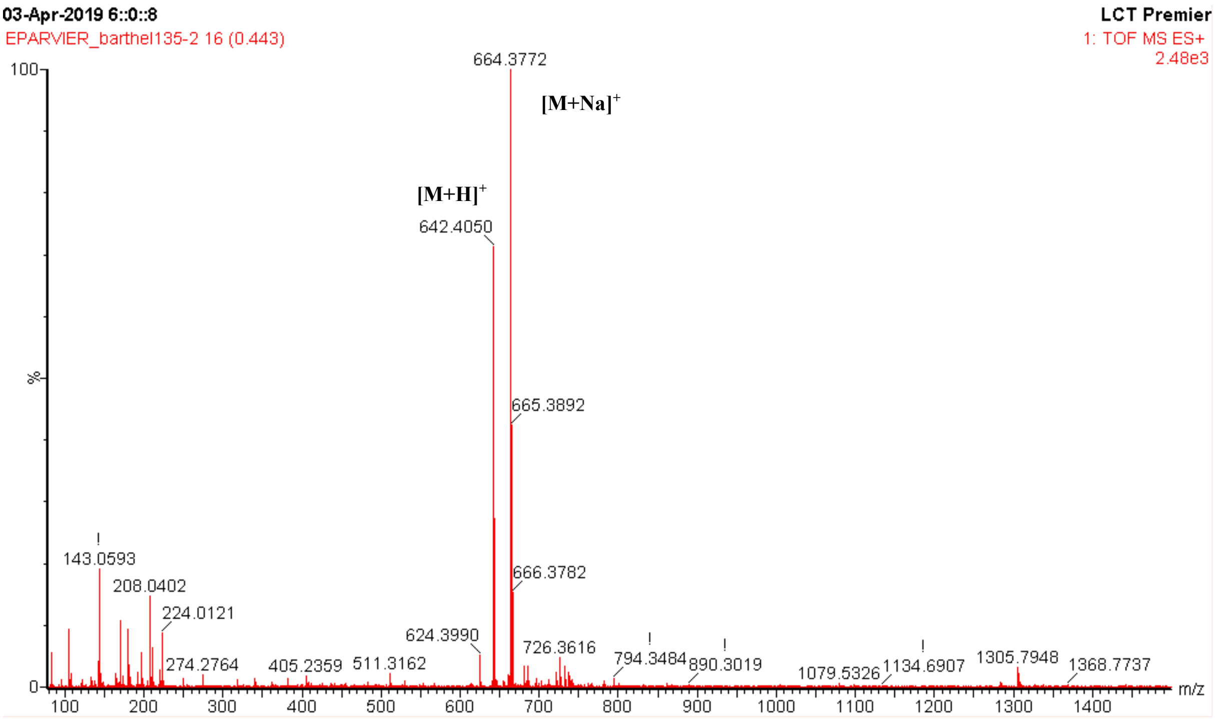

Supplementary Figure S14: Mass spectrum MS/MS of compound **2** – A: The enlargement of ion peak at *m/z* 155.1437 relevant to the acyl chain

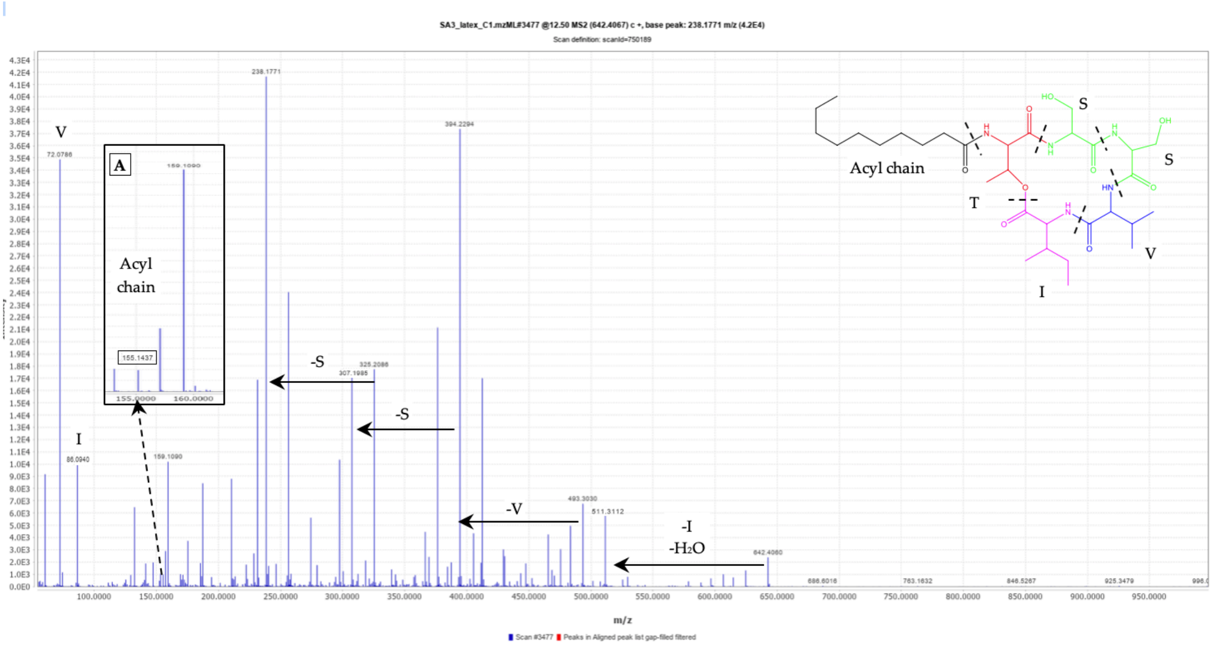

Supplementary Figure S15: ^{1}H NMR spectrum of compound **2** recorded at 500 MHz in CD_3CN

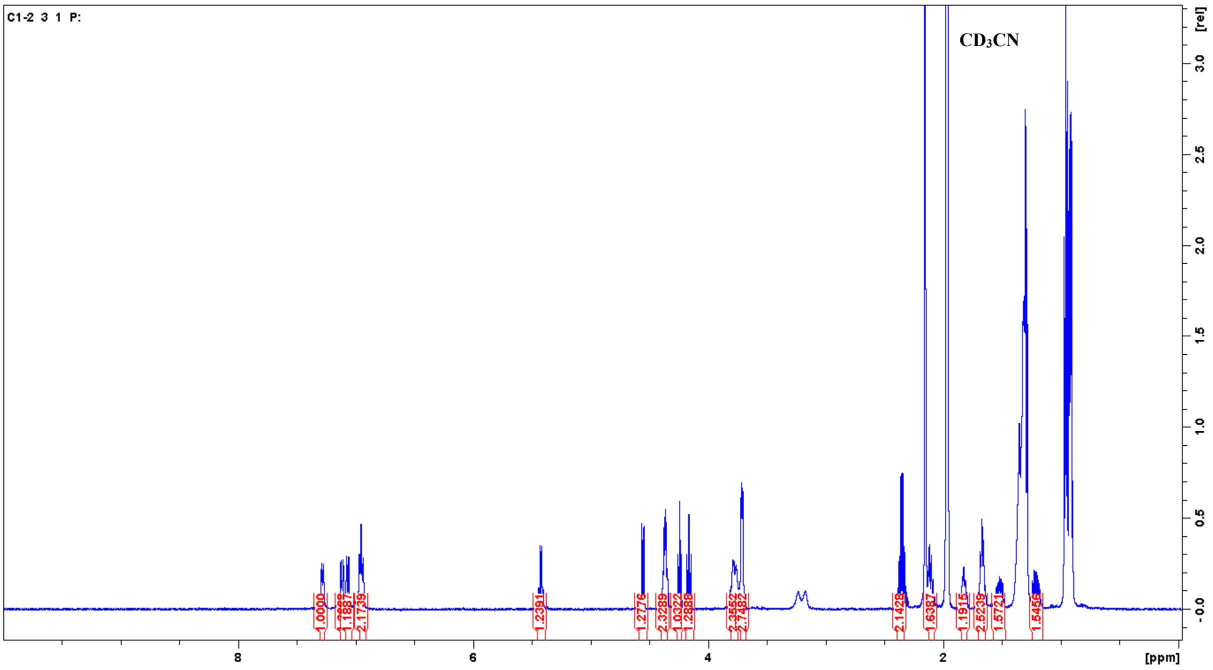

Supplementary Figure S16: ^{1}H-^{1}H COSY spectrum of compound **2** recorded at 500 MHz in CD_3CN

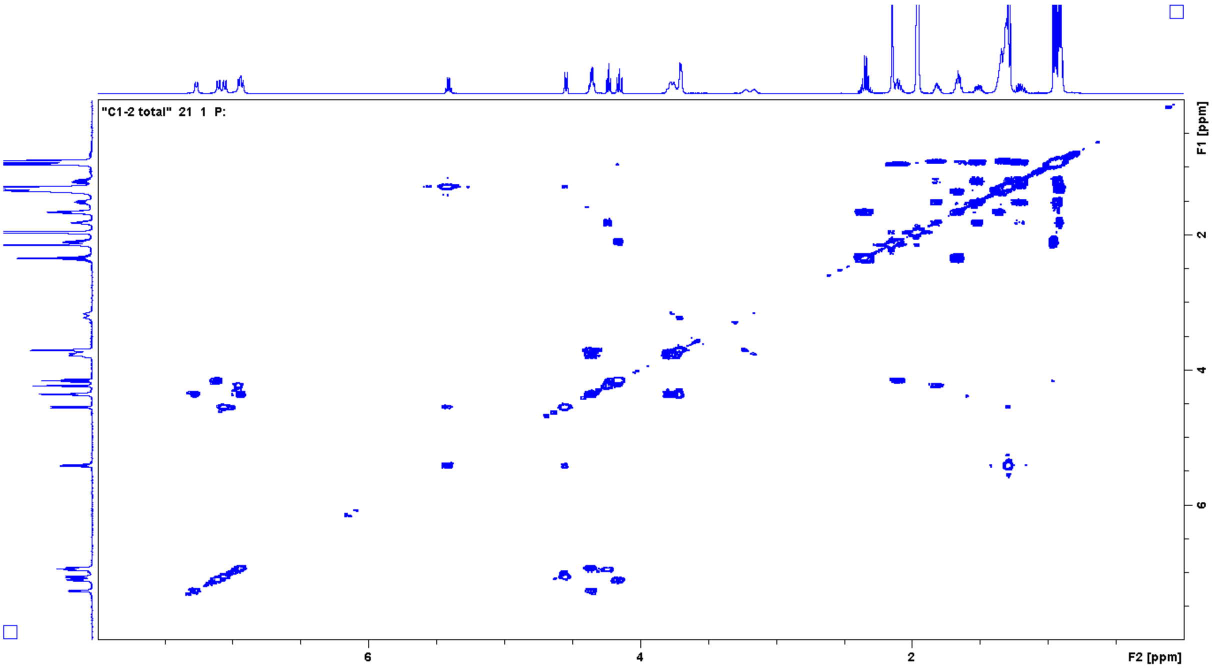

Supplementary Figure S17: ^{1}H-^{13}C HSQC spectrum of compound **2** recorded at 500 MHz in CD_3CN

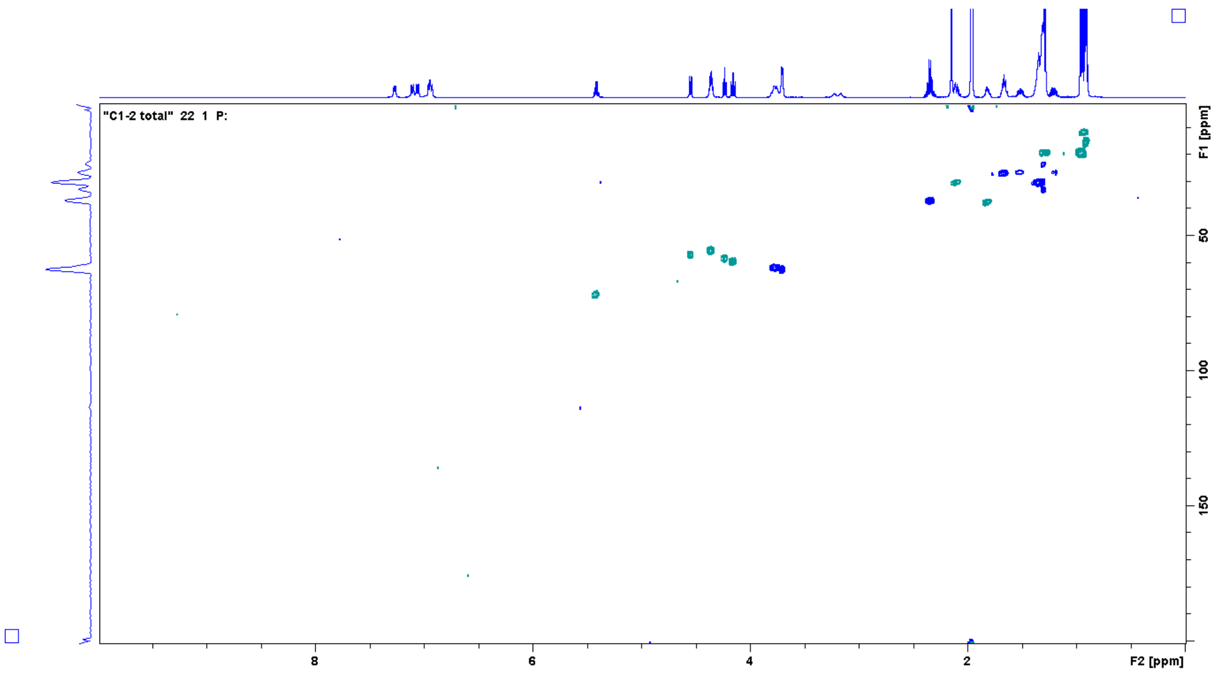

Supplementary Figure S18: ^{1}H-^{13}C HMBC spectrum of compound **2** recorded at 500 MHz in CD_3CN

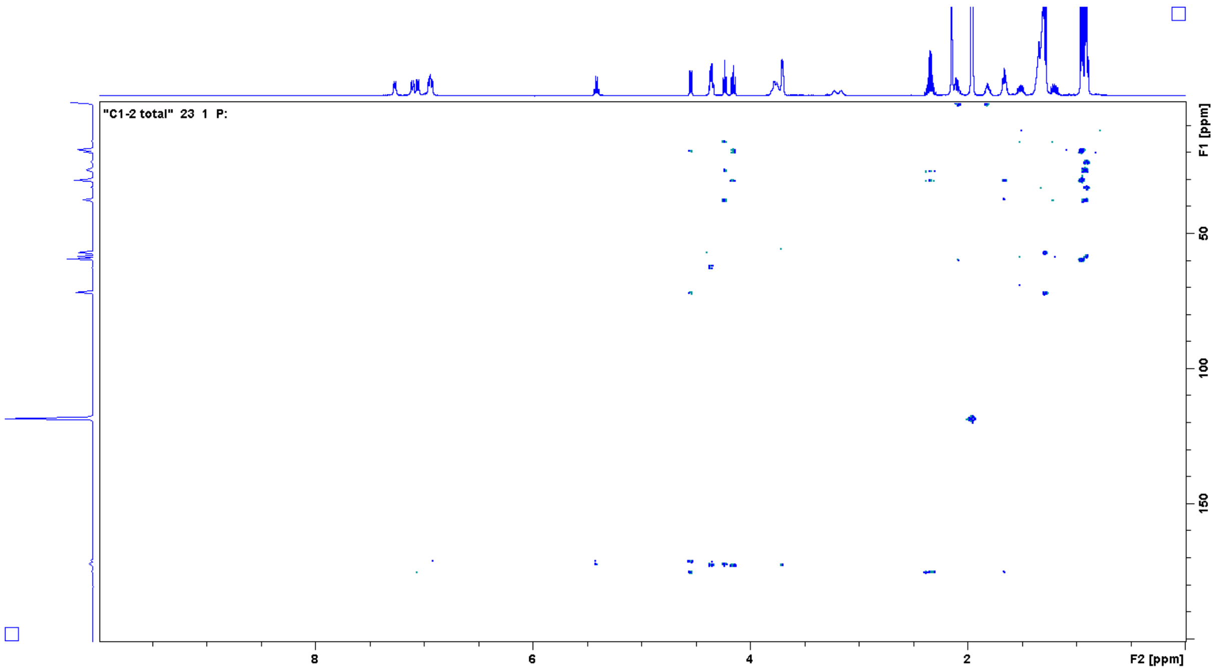

Supplementary Figure S19: Mass spectrum of compound **3**

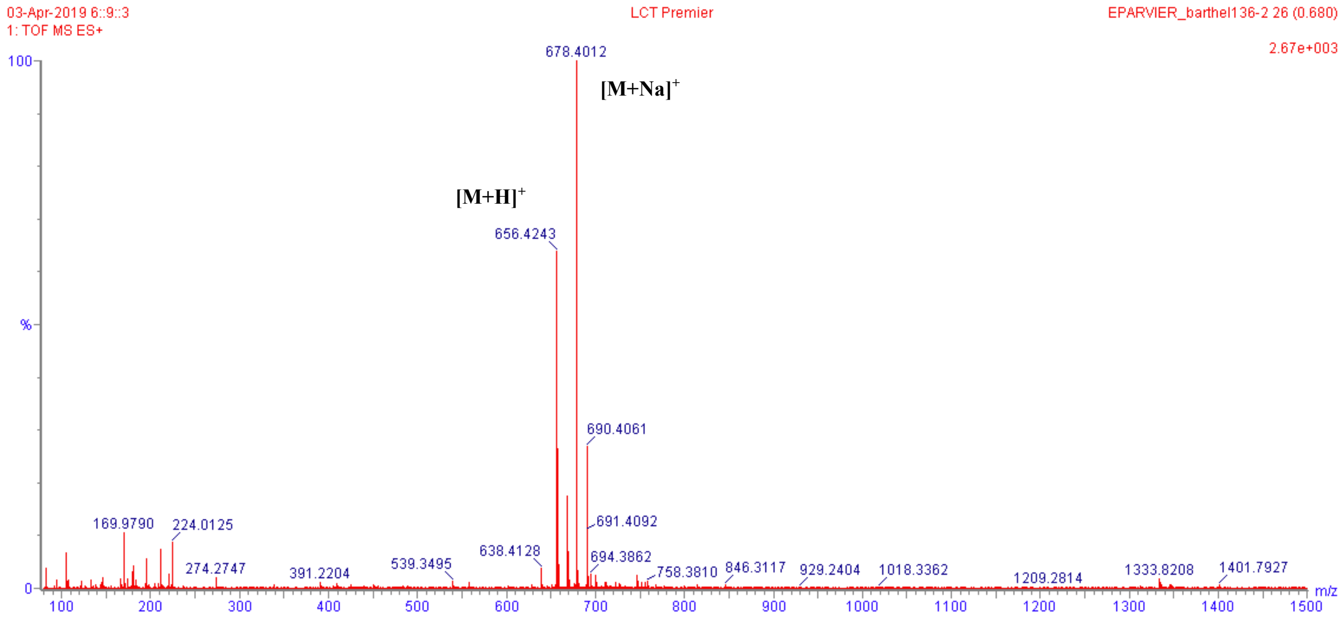

Supplementary Figure S20: Mass spectrum MS/MS of compound **3** – A: The enlargement of ion peak at *m/z* 183.1709 relevant to the acyl chain.

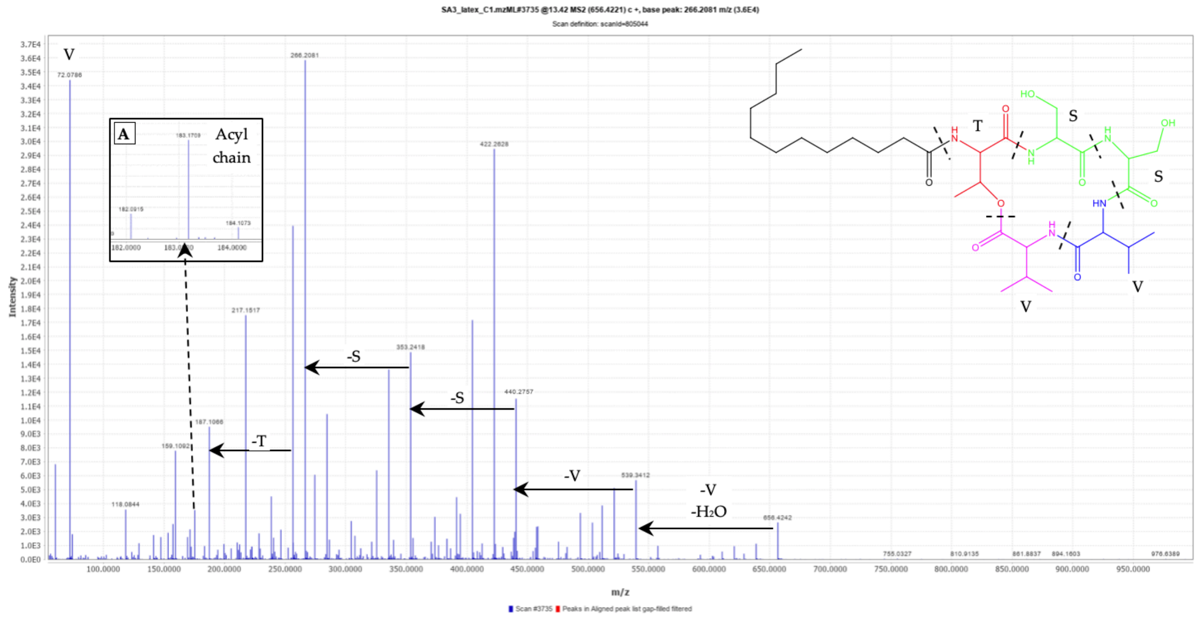

Supplementary Figure S21: ^{1}H NMR spectrum of compound **3** recorded at 500 MHz in CD_3CN

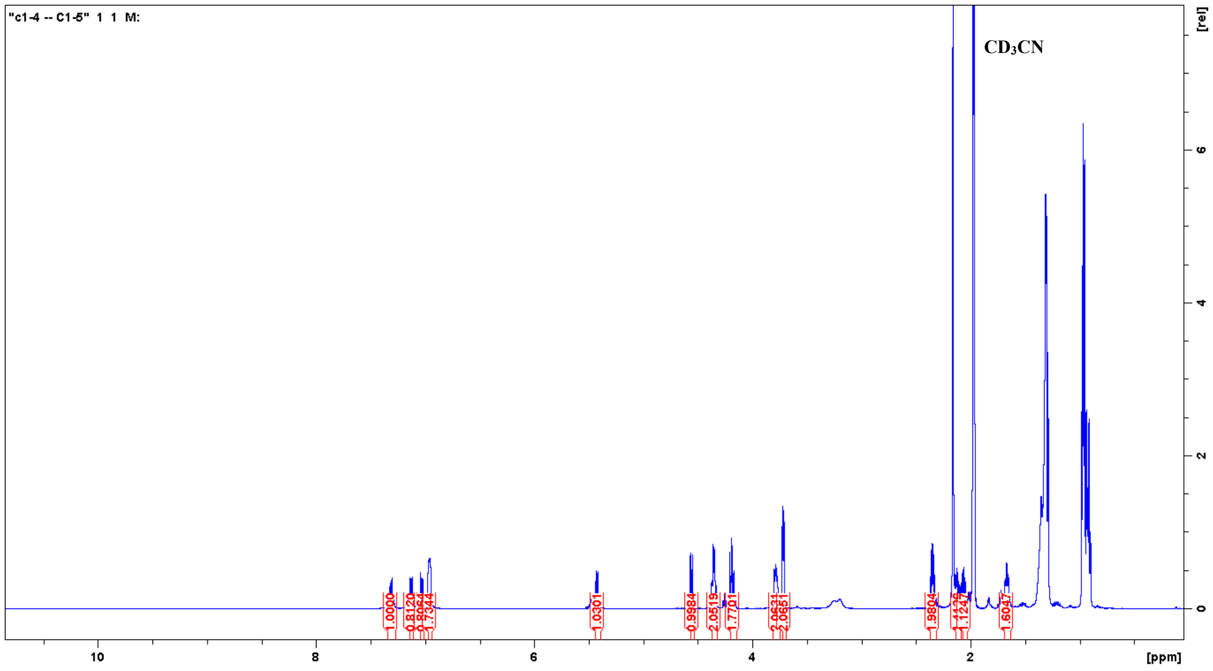

Supplementary Figure S22: ^{1}H-^{1}H COSY spectrum of compound **3** recorded at 500 MHz in CD_3CN

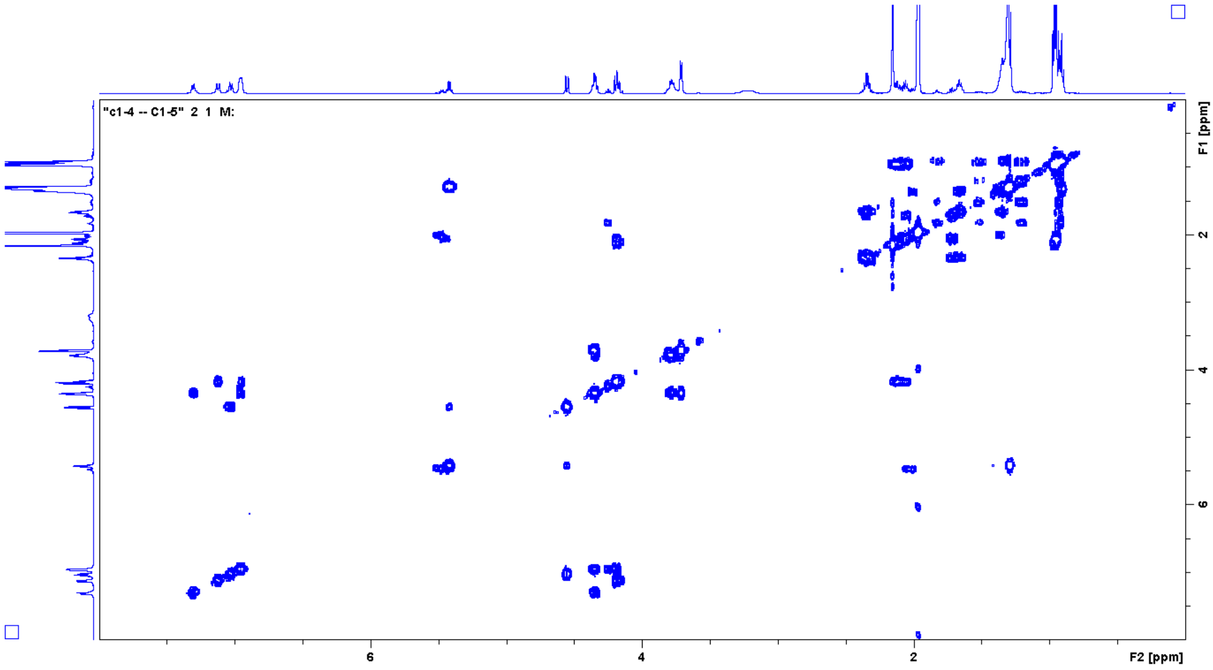

Supplementary Figure S23: ^{1}H-^{13}C HSQC spectrum of compound **3** recorded at 500 MHz in CD_3CN

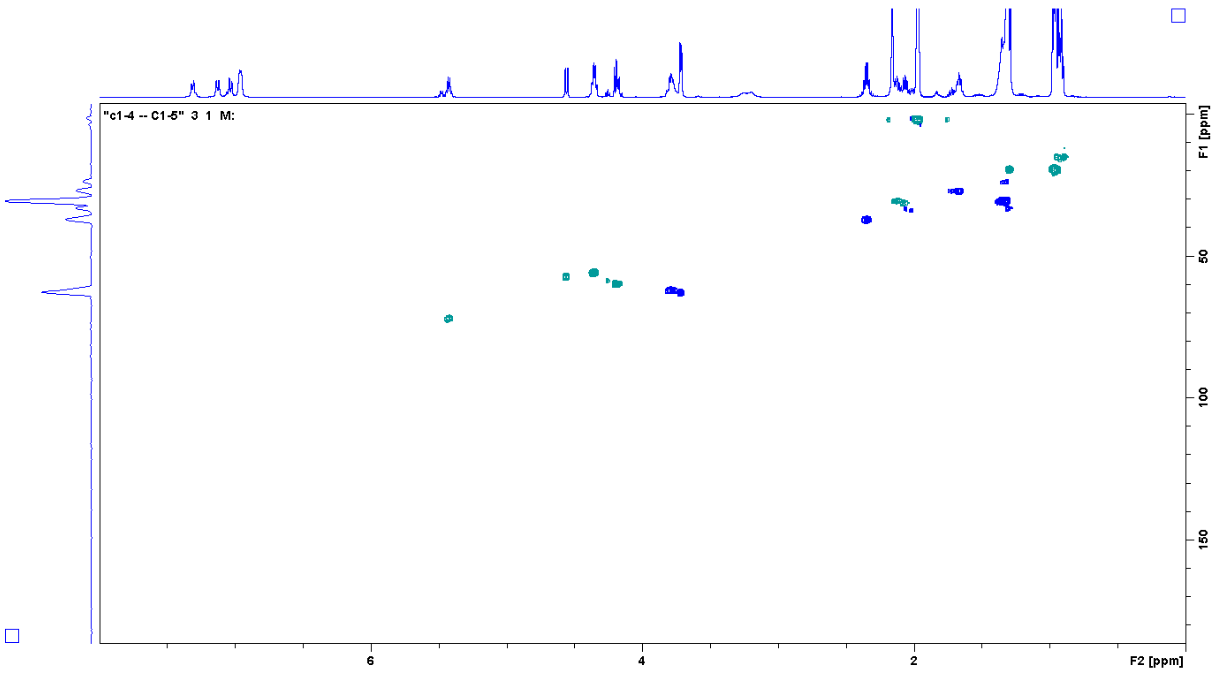

Supplementary Figure S24: ^{1}H-^{13}C HMBC spectrum of compound **3** recorded at 500 MHz in CD_3CN

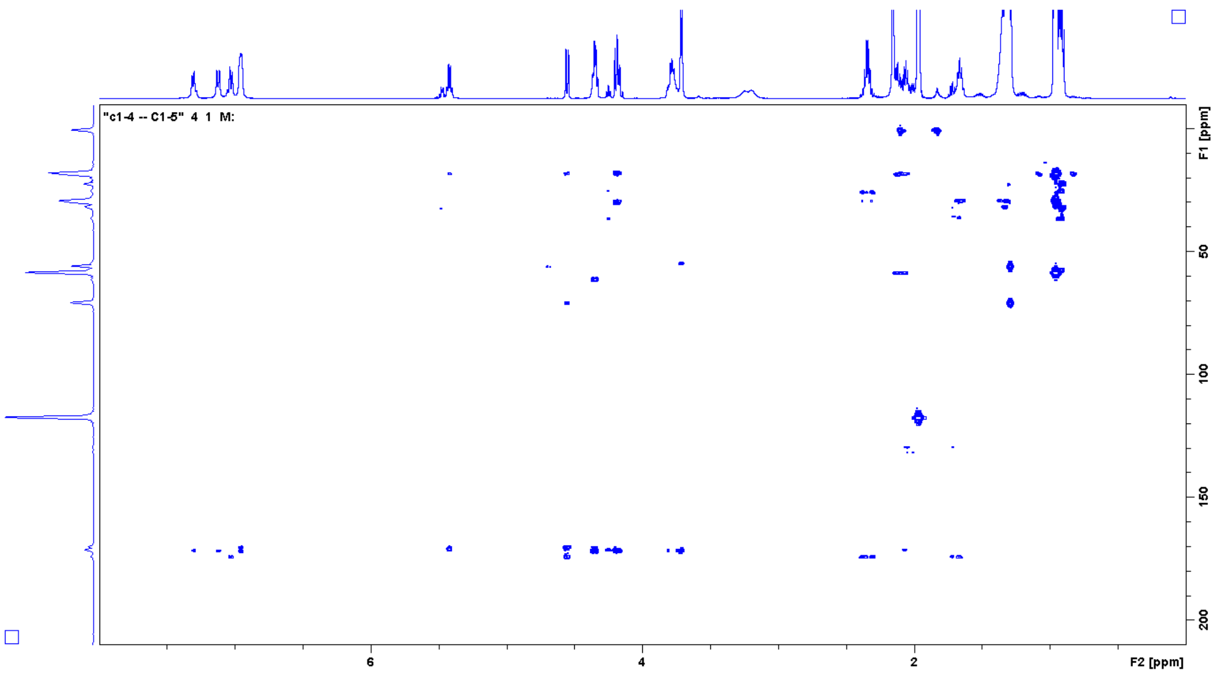

Supplementary Figure S25: Mass spectrum of compound **4**

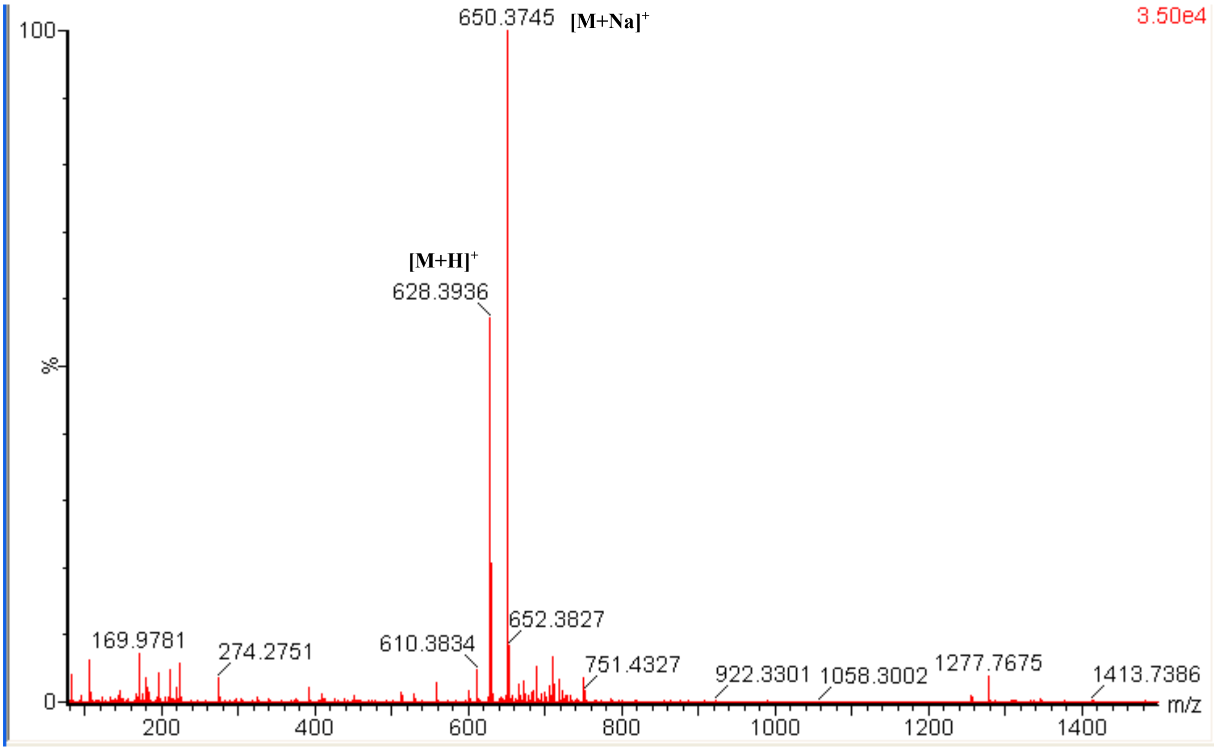

Supplementary Figure S26: Mass spectrum MS/MS of compound **4** – A: The enlargement of ion peak at *m/z* 155.1441 relevant to the acyl chain.

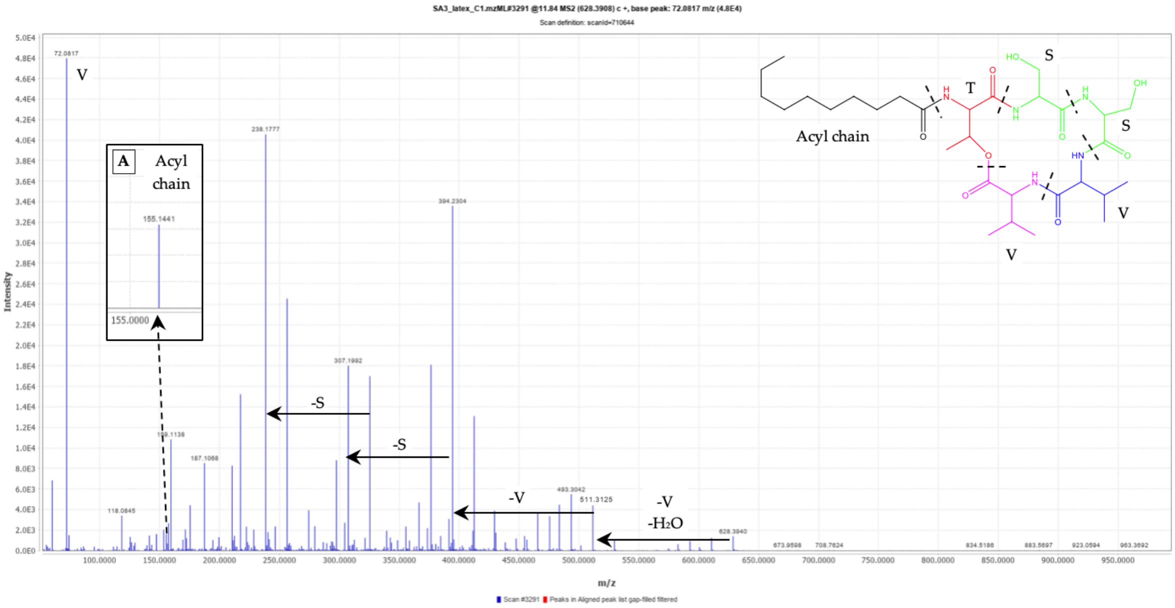

Supplementary Figure S27: ^{1}H NMR spectrum of compound **4** recorded at 500 MHz in CD_3CN

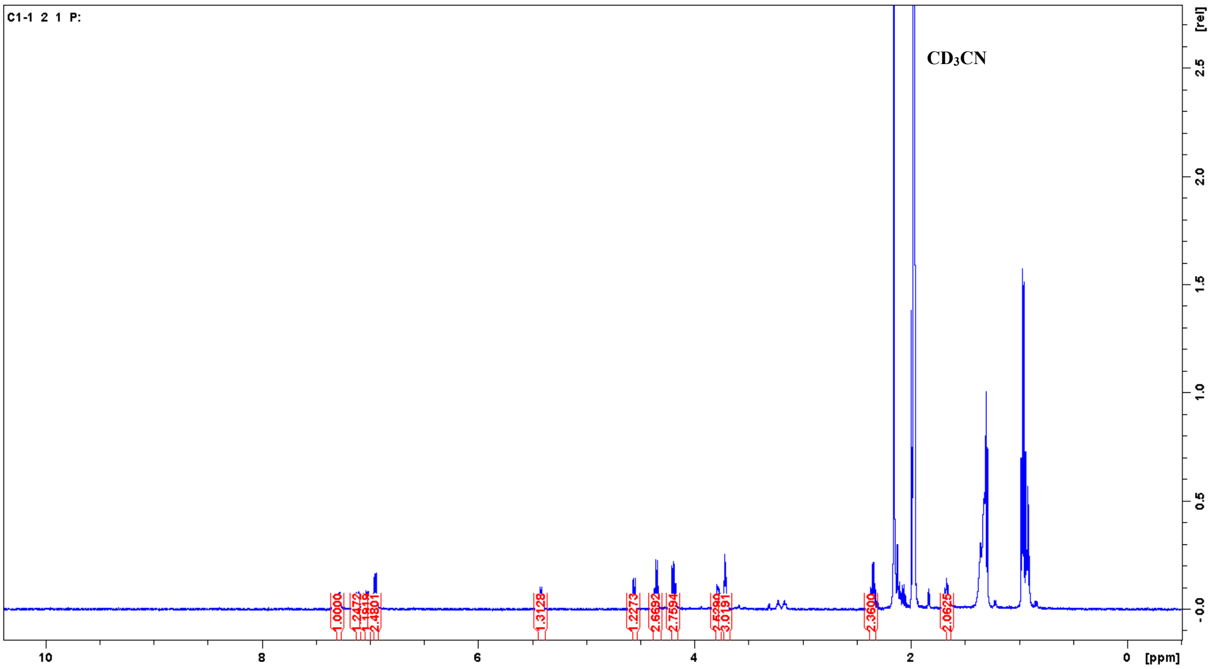

Supplementary Figure S28: Mass spectrum of compound **5**

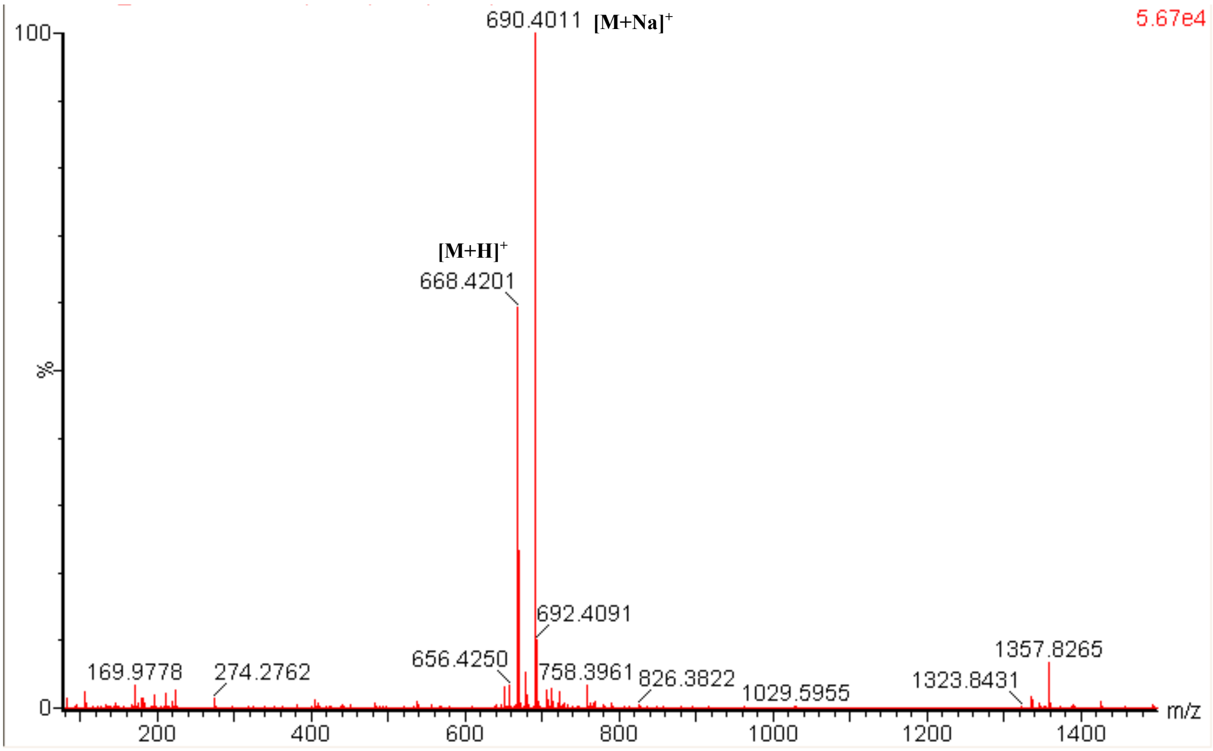

Supplementary Figure S29: Mass spectrum MS/MS of compound **5** – A: The enlargement of ion peak at *m/z* 181.1596 relevant to the acyl chain.

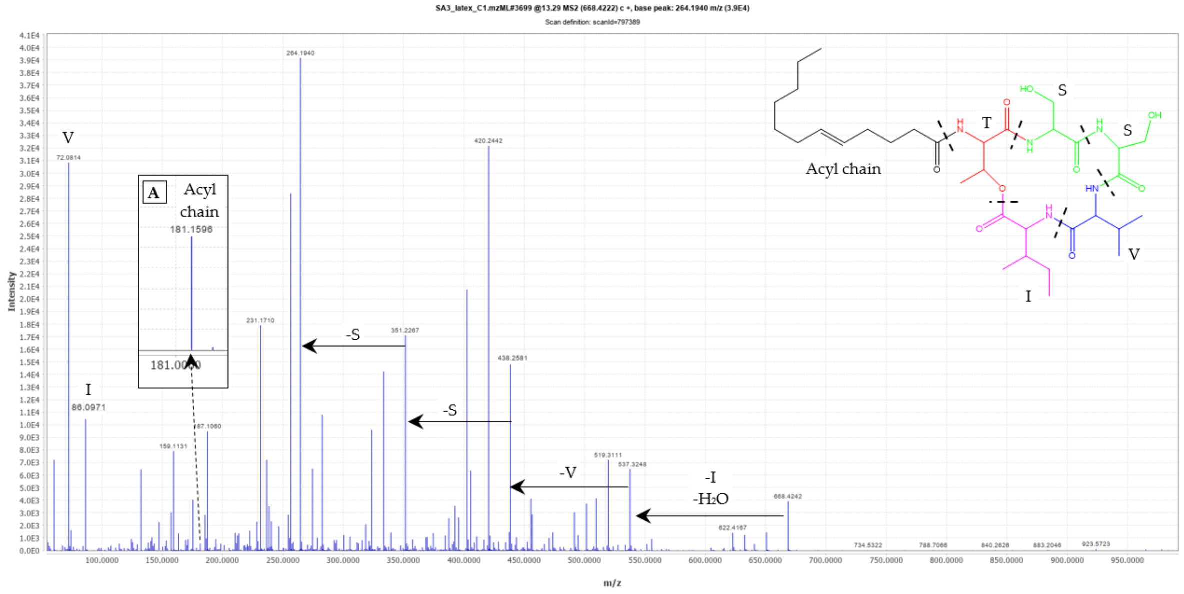

Supplementary Figure S30: ^{1}H NMR spectrum of compound **5** recorded at 500 MHz in CD_3CN

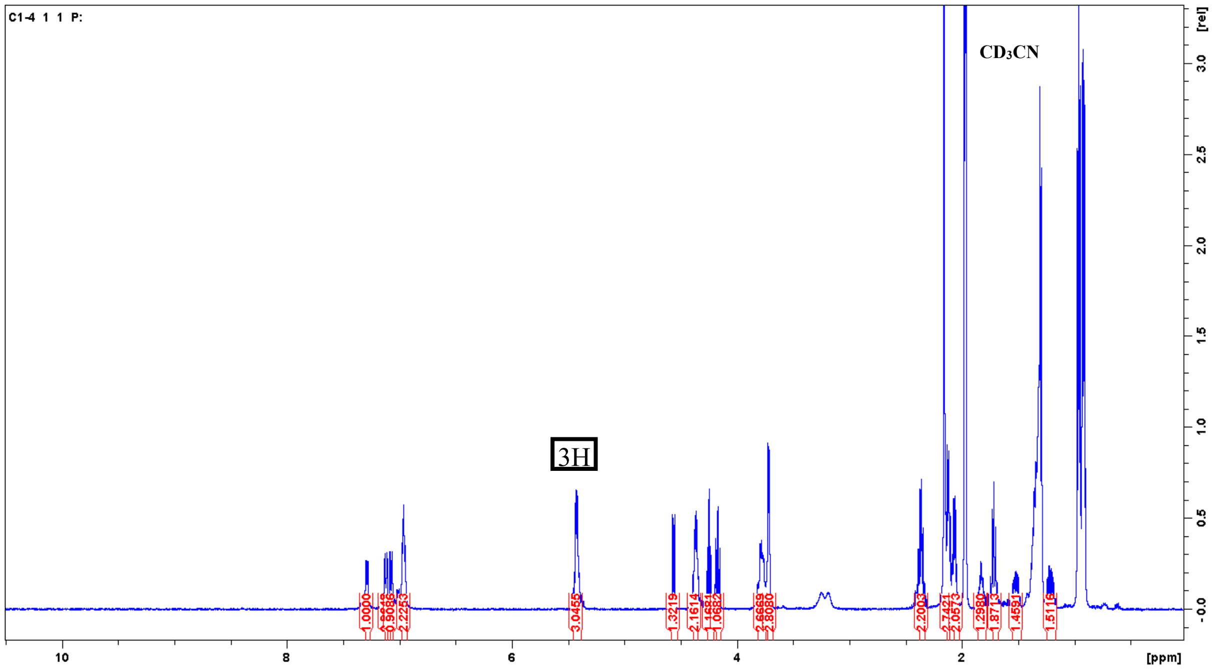

Supplementary Figure S31: ^{1}H-^{1}H COSY spectrum of compound **5** recorded at 500 MHz in CD_3CN

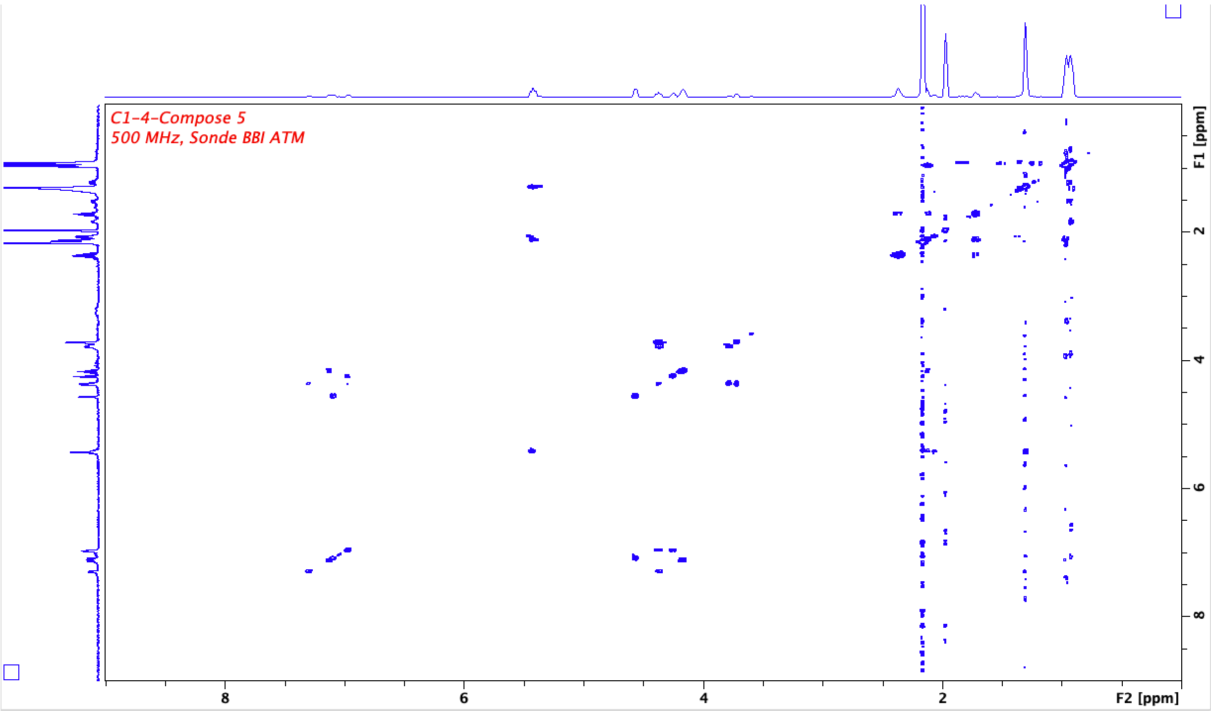

Supplementary Figure S32: ^{1}H-^{13}C HSQC spectrum of compound **5** recorded at 500 MHz in CD_3CN

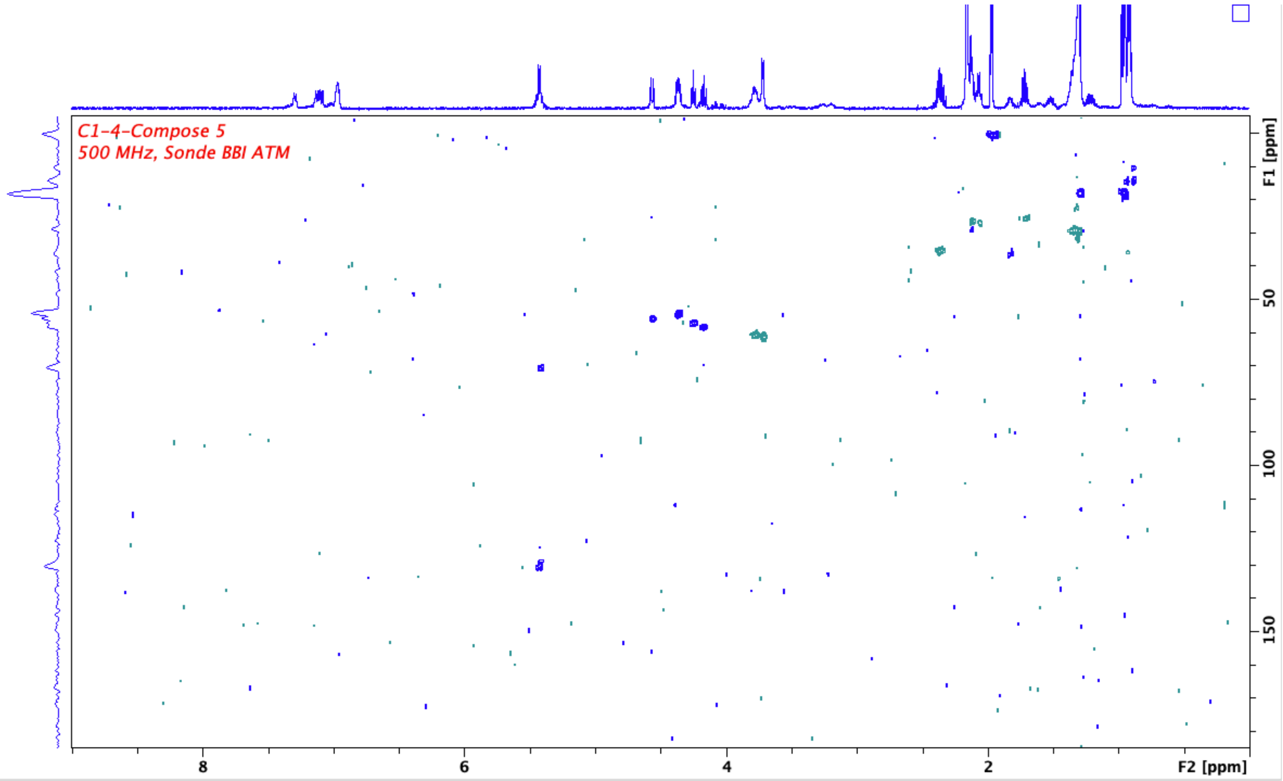

Supplementary Figure S33: ^{1}H-^{13}C HMBC spectrum of compound **5** recorded at 500 MHz in CD_3CN

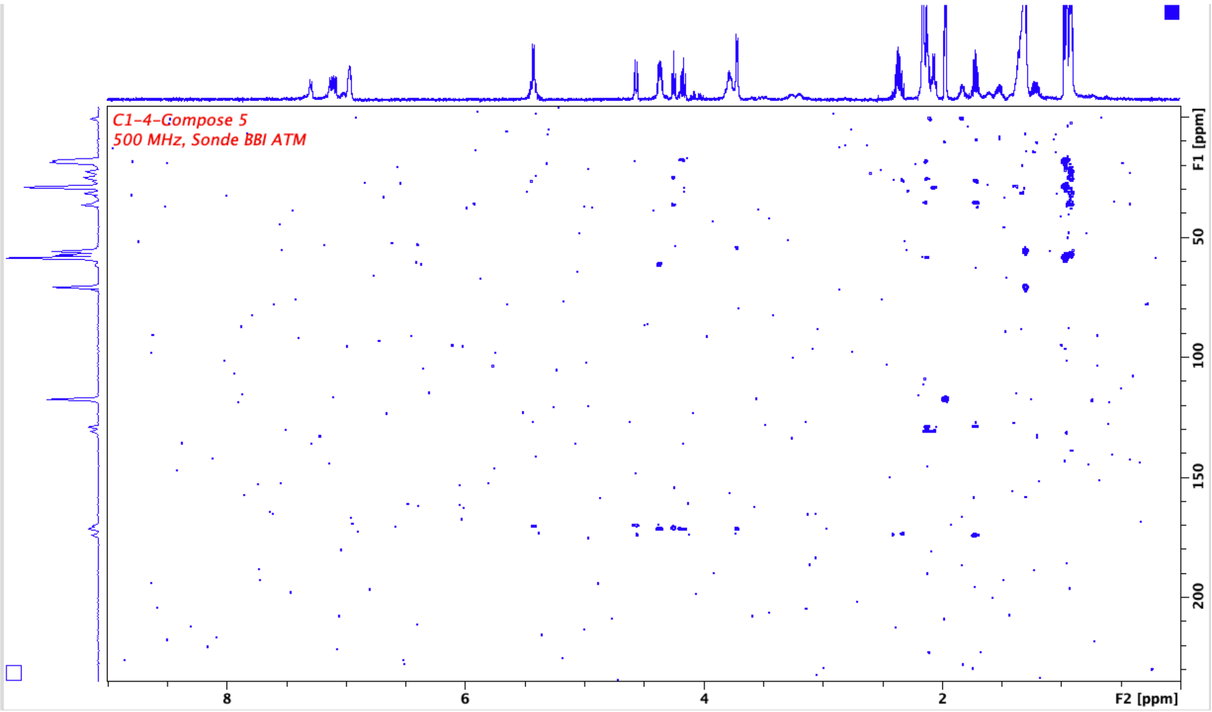

Supplement: Supplementary file 1 [file plants-09-00047-s001.pdf]
